# Host and habitat shape ectoparasite diversity on *Mastomys natalensis* and *Mastomys coucha* (Muridae)

**DOI:** 10.1017/S0031182024000714

**Published:** 2024-07

**Authors:** Alyssa J. Little, Conrad A. Matthee, Eddie A. Ueckermann, Ivan G. Horak, Cang Hui, Sonja Matthee

**Affiliations:** 1 Department of Conservation Ecology and Entomology, Stellenbosch University, Stellenbosch, South Africa; 2 Evolutionary Genomics Group, Department of Botany and Zoology, Stellenbosch University, Matieland, South Africa; 3 Unit for Environmental Sciences and Management, Potchefstroom Campus, North-West University, Potchefstroom, South Africa; 4 Department of Zoology and Entomology, Rhodes University, Makhanda, South Africa; 5 Department of Mathematical Sciences, Centre for Invasion Biology, Stellenbosch University, Matieland, South Africa; 6 Biodiversity Informatics Unit, African Institute for Mathematical Sciences, Muizenberg, South Africa

**Keywords:** ectoparasites, Epifaunistic species, habitat type, host sex, *Mastomys coucha*, *Mastomys natalensis*

## Abstract

*Mastomys natalensis* and *M. coucha* are commensal rodent species endemic to Africa. A recent taxonomic revision within *Mastomys* leaves the parasite–host list of *M. natalensis* questionable and that of *M. coucha* incomplete. The current study aimed to develop a better understanding of the ectoparasite diversity associated with the 2 distinct but closely related rodent species and to explore the influence of host and habitat type on ectoparasite infestations. Between 2014 and 2020, 590 rodents were trapped in 3 habitat types (village, agriculture and natural) across a wildlife-human/domestic animal interface. In total 48 epifaunistic species (45 ectoparasitic and 3 predatory) represented by 29 genera from 4 taxonomic groups (fleas, lice, mites and ticks) were recorded. Only 50% of the epifauna were shared between the 2 rodent species, with mites the most speciose taxon in both host species. The abundance of epifaunistic individuals, and also those of mites and fleas, were significantly higher on male *M. natalensis*, while ticks were significantly higher on reproductively active *M. natalensis*. For both rodent species, infestations by most epifaunistic taxa (on *M. natalensis*) and some taxa (on *M. coucha*) were significantly lower in the village as opposed to the less disturbed agricultural and natural habitat types. The study highlights the importance of host life history, even in closely related rodent species, in shaping parasite profiles and a loss of parasite diversity in more extreme anthropogenic habitats.

## Graphical abstract

**Figure:**
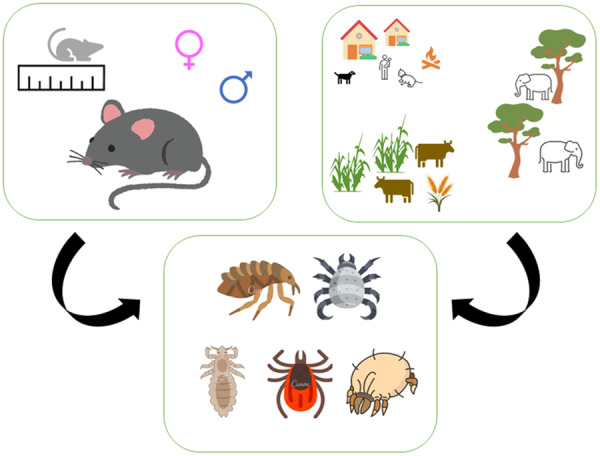

## Introduction

Rodentia is the largest mammalian order and have successfully colonized most of the globe (Wilson and Reeder, 2005). Their generally small size, vagility and adaptability enables them to occur in diverse habitats across the globe where they also encounter various parasites occurring in the external environments, in their nests and on the bodies of co-occurring hosts. For example, free-living immature life stages (larvae and/or nymphs) of most ixodid ticks and chiggers (trombiculid mites) attach to rodents when they move through vegetation, while lice are transferred between conspecifics through close body contact (e.g. during suckling, grooming and nest sharing) (Morand *et al*., 2006). Given the diverse life-history characteristics displayed by rodents (e.g. sociality, body size, habitat preference and nest types) it is expected that their parasite profiles will be influenced by life-history traits (Krasnov *et al*., 2010; Morand and Bordes, 2015). Indeed, higher parasite infestations are often associated with larger bodied hosts (providing more available niches and/or resources) (Kamiya *et al*., 2014; Esser *et al*., 2016) and high host population densities (providing more opportunity to encounter parasites) (Arneberg, 2001; Altizer *et al*., 2003). In addition, rodents that are geographically widespread (occur in multiple vegetation types) encounter diverse parasite species mainly due to vegetation type associated parasite distributions (i.e. distance decay in species similarity) (Spickett *et al*., 2017; Wells *et al*., 2018; Stevens *et al*., 2022).

Geographically widespread rodent species are often opportunistic in nature and take advantage of alternative resources available in anthropogenic habitats to the extent that they become pests (Drazo *et al*., 2008; Makundi and Massawe, 2011; Welegerima *et al*., 2020). Habitat transformation generally results in a change in the microclimatic conditions due to change in vegetation structure and reduced vegetation cover (Gehlhausen *et al*., 2000; Newman *et al*., 2013). Consequently, variation in the microclimatic conditions between habitat types (e.g. natural and transformed habitat types) is documented to affect parasite occurrence and infestation levels (Lorch *et al*., 2007; Froeschke *et al*., 2013; Froeschke and Matthee, 2014; van der Mescht *et al*., 2016). The role of habitat-associated factors in shaping parasite infestations is mainly related to the susceptibility of free-living life stages to desiccation (Krasnov *et al*., 2001*a*, 2001*b*; Herrmann and Gern, 2013; van der Mescht *et al*., 2013). Parasite taxa with free-living life stages (nematodes, fleas, mites and ticks) are particularly more sensitive to the microclimatic conditions (e.g. air temperature and relative humidity) compared to parasite taxa, such as lice, where all life stages occur permanently on the host's body (Krasnov *et al*., 2010; Viney and Cable, 2011; Härkönen *et al*., 2013).

To further investigate the role of abiotic factors (the environment) and host life history on parasite profiles of rodents, we herein study 2 multimammate mice species, *Mastomys natalensis* and *M. coucha*. In the 1970's, *M. natalensis* underwent taxonomic revision and a second species, *M. coucha*, was recognized based on differences in chromosome numbers, haemoglobin patterns, mtDNA sequencing, and subtle differences in the morphology of the cranium (Gordon, 1978; Green *et al*., 1980; Granjon *et al*., 1997). The 2 rodent species are widely distributed habitat generalists that overlap in occurrence in the north-eastern and eastern summer rainfall region (Savanna and Grassland biomes) in South Africa (Skinner and Chimimba, 2005; Monadjem *et al*., 2015). However, based on previous studies it appears that *M. natalensis* are more commensal (associated with houses) (Brouat *et al*., 2007; Mulungu *et al*., 2011, 2013), and that *M. coucha* could prefer less disturbed habitats. The latter may be mainly attributed to the higher breeding success of *M. natalensis* on a poor quality diet when compared to *M. coucha* (Jackson and van Aarde, 2004). *Mastomys* species are prolific breeders, live in family groups (Isaacson, 1975; Leirs *et al*., 1996*a*, 1996*b*) and can attain high population densities in anthropogenic habitats (Makundi and Massawe, 2011). In particular, *M. natalensis,* is regarded as an agricultural pest (Singleton *et al*., 2003; Prakash, 2018) where they frequently utilize abandoned burrows of other rodent species (Veenstra, 1958). *Mastomys natalensis* is also a reservoir host for disease causing pathogens such as Lassa virus that causes haemorrhagic fever, and *Yersinia pestis* which is the causative agent for bubonic plague (Isaacson, 1975; Singleton *et al*., 2003; Achtman *et al*., 2004; Lecompte *et al*., 2006). From a disease perspective, it is important to realize that the movement of commensal rodent species between habitat types and higher densities recorded in anthropogenic habitats, creates novel opportunities for parasite exchange and may pose a disease risk to domestic animals and humans (Lecompte *et al*., 2006; Brettschneider *et al*., 2012).

Although the ecology and taxonomy of *M. natalensis* and *M. coucha* is relatively well studied, information regarding their ectoparasite profiles is limited and biased towards parasite–host lists originally described for *M. natalensis* (Zumpt, 1961; Isaacson, 1975; Ledger, 1980; Segerman, 1995; Horak *et al*., 2018). Given the taxonomic revision of the host genus, and the fact that the 2 rodent species are cryptic, it is essential that the ectoparasite profile of *M. natalensis* is re-assessed and an ectoparasite profile established for *M. coucha.* More recent empirical studies documented a rich ectoparasite diversity associated with widely distributed rodents in South Africa (e.g. Matthee *et al*., 2007, 2010; Froeschke *et al*., 2013; Archer *et al*., 2014; Fagir *et al*., 2014, 2015; Stevens *et al*., 2022; Smith *et al*., 2023) and made a considerable contribution in updating parasite lists for these rodents. Several of the ectoparasite taxa recorded in these studies are known vectors for disease-causing pathogens such as *Y. pestis* (causative agent of plague) and *Rickettsia africae* (causative agent of African tick-bite fever) (Achtman *et al*., 2004; Ledger *et al*., 2022). To date only a few studies have explored the ecological factors (e.g. host and environment) that shape parasite infestations in South Africa (Froeschke *et al*., 2010, 2013; Froeschke and Matthee, 2014; van der Mescht *et al*., 2016; Smith *et al*., 2023). Although these studies confirm the importance of the host, habitat type and climate in shaping parasite infestations more studies on rodents with diverse life histories are needed before general patterns can be established.

The overall aim of the study was to develop a better understanding of the ectoparasite diversity associated with the 2 distinct but closely related rodent species and to explore the role of host and habitat type in shaping ectoparasite infestations. The objectives of the study were: (1) Record the ectoparasite diversity associated with *M. natalensis* and *M. coucha* across a wildlife-humans/domestic animal interface, and (2) Explore the relationship between ectoparasite infestations, and host (sex, breeding state and body size) and habitat (village, agriculture and natural) factors in both rodents. Given the close evolutionary relationship between the rodents studied herein, it is predicted that the ectoparasite profile will largely overlap between *M. natalensis* and *M. coucha*. Further, it is predicted that ectoparasite infestations will be related to, (i) host sex, with higher infestations associated with larger-bodied male hosts, and (ii) habitat type, where ectoparasite taxa with free-living life stages (fleas, mites and ticks) will respond more strongly to habitat transformation.

## Materials and methods

### Study area

**Figure 1.**
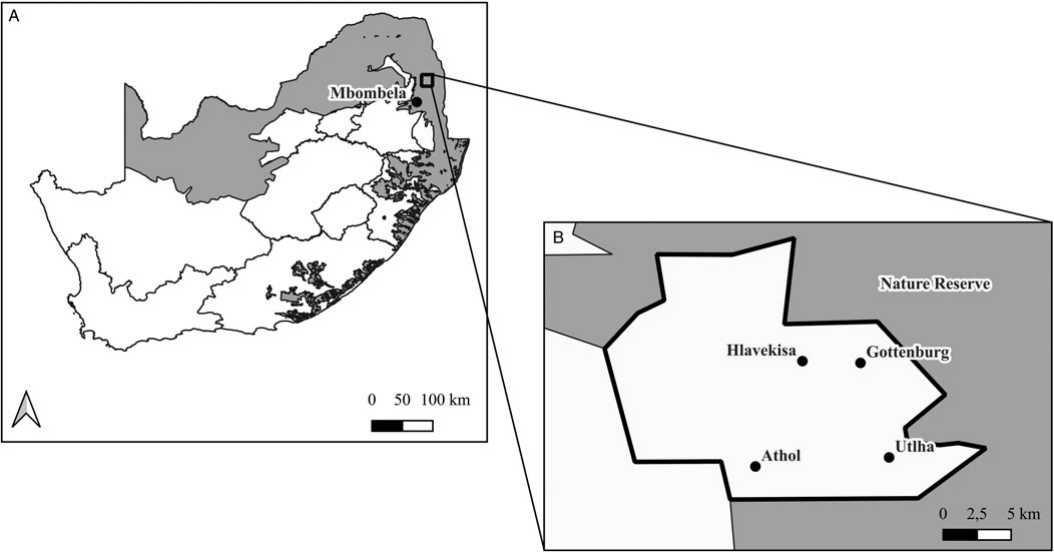
Study area where *Mastomys natalensis* (*n* = 375) and *Mastomys coucha* (*n* = 215) were trapped across a wildlife-human/domestic animal interface in Mpumalanga, South Africa. (a) Savanna biome (grey) in South Africa and the position of the sampling area (square on map). (b) position of the villages and the nature reserve.

The study was conducted across a wildlife-human/domestic animal interface, located in the Mnisi rural community situated in the north-eastern Savanna biome of South Africa. The community comprises of several small villages that each have their own communal cattle grazing area and subsistence crop fields (also referred to as agriculture). Approximately 75% of the boundary surrounding the Mnisi rural community is shared with adjacent fenced nature reserves (Fig. 1). Rodents were trapped in the Manyeleti nature reserve (24°35′0.1″ S, 31°25′56″ E) and in 4 villages and their respective crop fields (Gottenburg 24°38′01″ S, 31°25′19″ E; Hlavekisa 24°37′51″ S, 31°22′42″ E, Athol 24°42′29″ S, 31°20′43″ E and Utlha 24°50′14″ S, 31°02′45″ E) in 2014, 2015 and again in 2019 and 2020. The villages were >5 km apart from each other. Small vegetable patches, cattle and other domestic animals can be found on the property (in the village). Crop fields were planted with seasonal crops that were fenced with a combination of wire and dried tree branches that were stacked to form a fence. The crop fields were situated on the edge of the villages and often occurred between the village and nature reserve. Cattle could be found in the crop fields during the dry season. The nature reserve comprised of pristine natural Savanna vegetation and biome-associated wildlife species.

### Rodent trapping and handling

Sherman-type live traps and Tomahawk live traps were used to trap rodents across 3 different habitat types namely, village (transformed), agriculture (semi-transformed), and natural (undisturbed). Rodents were trapped at 3 villages and their respective crop fields in spring (August–October) of 2014 and 2015, and once in summer (January) of 2015 and at 4 villages and their respective crop fields in spring (August–October) 2019 and 2020. A standardised trap design was followed every year, and each locality was only trapped once per trap session. Traps were placed at 10 m intervals along an 80 m-trap line that was replicated (3–4 times) at each sampling locality. The traps were left out for 3–4 days per locality. In the village, traps were set in and around the houses. In the agricultural habitat traps were placed along the fence of the crop fields, whereas in the nature reserve traps were set in trap lines in the natural vegetation. A mixture of oats and peanut butter was used as bait. Traps were checked twice daily and closed during the heat of the day (10:00–15:00). Targeted rodents were removed from the traps, individually placed into labelled plastic bags and euthanized with Isoflurane. All non-target rodent species were recorded and released at the trap sites. Targeted rodent species were frozen at −20°C to kill all ectoparasites. The rodents were initially morphologically identified to genus level using field guides (Stuart and Stuart, 2007) and thereafter species were confirmed molecularly using a species-specific mtDNA cytochrome-b multiplex PCR (Bastos *et al*., 2005). For each rodent, their sex, reproductive state (breeding: males having a scrotum and females having a perforated vagina; or non-breeding: males with no visible scrotum and females with no perforated vagina), body weight, total-, tail- and hind foot- length was recorded. Rodents were dissected to further confirm rodent sex and reproductive state.

### Laboratory procedures

Prior to ectoparasite removal rodents were thawed. All ectoparasites (fleas, lice, mites and ticks) and a subsample of trombiculid mites (chiggers) were systematically removed with fine point forceps while examining the body of the rodent under a Zeiss Stemi DV4 stereomicroscope (Carl Zeiss Light Microscopy, Göttingen, Germany). In the case of chiggers, the parasitope (region on the body where chiggers occurred) was recorded for each subsample. Ectoparasites were counted and placed into individual tubes containing 100% ethanol. All fleas (males and females) were counted, however only male individuals were available for species level identification and counts per species, as female fleas were used for a separate project and could not be identified to species level. The immature life stages of individual louse species remained undifferentiated and was reported as nymphs and counts presented per species. Fleas were mounted (in Canada balsam) as described by Segerman (1995) and van der Mescht *et al*. (2013). Lice and mites were cleared in lactic acid and mounted in Hoyer's or PVA (polyvinyl alcohol) on microscope slides. A subsample of the lice was kept for molecular examination. Chiggers were directly slide mounted in Hoyer's or PVA mounting medium. Ticks remained in 100% ethanol for morphological identification. Identification of fleas, lice, mites and chiggers was done using a Leica DM1000 light microscope (Leica Microsystems GmbH, Wetzlar, Germany) and ticks were identified with a Leica MZ75 high-performance stereomicroscope (Leica Microsystems GmbH, Wetzlar, Germany). All ectoparasites were morphologically identified to species level where possible using taxonomic reference literature; fleas (Segerman, 1995); lice (Johnson, 1972; Ledger, 1980; Durden and Musser, 1994); mites (Till, 1963; Herrin and Tipton, 1975); chiggers (Zumpt, 1961; Stekolnikov, 2008, 2018) and ticks (Walker *et al*., 2000; Horak *et al*., 2018). In a few cases the differentiation between *Hoplopleura intermedia* and *H. ismailiae* louse individuals was troublesome and are referred to as *H. intermedia/ismailiae*.

### Data analysis

The relative host density, for the 2 rodent species, was estimated on trapping success (%) (number of trapped animals divided by the number of trap nights multiplied by the number of traps) (Froeschke *et al*., 2013). For descriptive statistics on epifaunistic infestations per rodent species, rodent and epifaunistic data were pooled per locality within each of the habitat types (village, agriculture and natural) for all sampling years (2014–2020 for all taxa except mites, as species-level abundance data for mites was only available for 2019–2020) and seasons. For each rodent species we divided the epifauna into higher taxonomic groups (fleas, lice, mites and ticks) and pooled the different life stages (i.e. larvae, nymphs, males and females) within the respective taxa. The mean abundance (total epifaunistic abundance divided by the number of hosts) and prevalence (% of hosts infested) were calculated following Bush *et al*. (1997). Chigger prevalence was calculated using presence/absence data from 2019–2020, as chigger species identification per rodent species was only available for this sampling period.

To explore the relationship between epifaunistic infestations, host and habitat factors the following approach was used: total counts of epifaunistic individuals for a given taxon (overall epifauna, fleas, lice, ticks and mites) were calculated for 2014–2020 (excluding January 2015) and species richness per taxon (overall epifauna, fleas and mites) was calculated for 2019–2020 on an individual host (i.e. infracommunity). Only rodents with confirmed reproductive state were used for regression analyses, which meant that a reduced sample size was used for these analyses. Epifaunistic count data (excluding chiggers) was modified (log + 1 transformed) prior to analyses as the data was highly skewed with an excess of zero's. All models were fit to examine the influence of host-related factors (sex, reproductive state, interaction between reproductive state and sex and body size (tail length as proxy)), habitat type (village, agriculture, natural) and sampling year on the total count for each ectoparasite taxon and species richness of each host species. A generalised linear model (GLM) was constructed for the overall epifauna and mite counts following a Poisson distribution for both rodent species (note, although a negative binomial distribution is often used for ectoparasite data, our preliminary analysis suggests a stronger support to the Poisson distribution). To account for the large number of zero's in flea, tick and louse counts, a zero-inflated Poisson (ZIP) and a zero-inflated negative binomial (ZINB) model were generated from ‘pscl’ package in R (R Core Team, 2023). The methodology and mathematics of the ZIP and ZINB models can be found in (Zeileis *et al*., 2008; Zuur *et al*., 2009; Zuur and Ieno, 2016). The ZIP or ZINB models, were compared using a Likelihood Ratio Test (LRT).

The relationship between epifaunistic, flea, mite species richness and host and habitat related factors were based on individual hosts of each rodent species. Species richness was not informative for lice (as the taxon was dominated by 1 or 2 louse species) and for ticks (due to low infestations: 4–5 individuals during the 2019–2020 sample period) for both rodent species. For the regression analyses of epifaunistic, flea and mite species richness a GLM with a Poisson distribution was used. A backward model selection was considered for all regression analyses (count and species richness), using a ‘step’ function for all models, whereby the models with the lowest Akaike information criterion (AIC) were presented as the final selected models (Burnham and Anderson, 2002; Snipes and Taylor, 2014). All statistical analyses were performed in R version 4.2.0 (R Core Team, 2023).

## Results

### Rodent density

**Figure 2.**
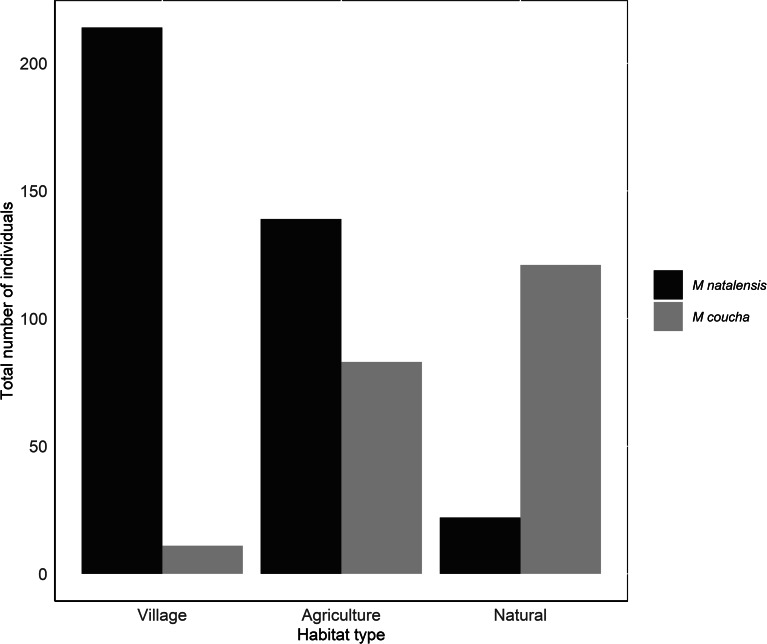
The total number of *Mastomys natalensis* (*n* = 375) and *Mastomys coucha* (*n* = 215), trapped in three habitat types across a wildlife-human/domestic animal interface in Mpumalanga, South Africa (2014–2020).

In total 375 *M. natalensis* and 215 *M. coucha* individuals were trapped (Supplementary Table 1). The number of individuals trapped, and their relative densities varied between habitat types for both rodent species (Supplementary Table 2; Fig. 2). In particular, the total abundance of *M. natalensis* was higher in the village and agricultural habitat types compared to the natural habitat. In contrast, the total abundance of *M. coucha* was higher in the natural and agricultural habitat types compared to the village habitat type (Fig. 2).

### Epifaunistic diversity

A total of 10 420 epifaunistic individuals (parasitic and non-parasitic) were recorded of which 5053 were recorded on *M. natalensis* and 5367 on *M. coucha* during the sampling period. Overall, 45 ectoparasitic and 3 non-parasitic species (predatory mites) were recorded on *M. natalensis* and *M. coucha* (Supplementary Table 3). Mites (excluding chiggers) were the most speciose taxon (23), followed by chiggers (8), fleas (7), ticks (6) and lice (4) (Supplementary Table 3). The 2 rodent species shared 23 of the 48 epifaunistic species (Supplementary Table 3). A larger number of epifaunistic species were recorded on *M. natalensis* (40) compared to *M. coucha* (31) (Supplementary Table 3).

Five flea species were recorded on *M. natalensis* and 6 species on *M. coucha*. However, fleas were more prevalent on *M. natalensis* (46.93%) compared to *M. coucha* (35.81%). *Xenopsylla brasiliensis* and *X. frayi* were the most prevalent fleas on both rodent species (Supplementary Tables 4 and 5). *Echidnophaga gallinacea* occurred on both rodent species, but the total abundance was higher on *M. natalensis* (9), while only 1 individual was recorded on *M. coucha*. Three louse species were recorded on *M. natalensis* and 2 on *M. coucha*. Lice were less prevalent on *M. natalensis* (33.33%) in comparison to *M. coucha* (54.42%). *Hoplopleura intermedia* was the most prevalent louse on both rodent species. *Polyplax biseriata* was only recorded on *M. coucha*, while *H. intermedia/ismailiae* and *P. spinulosa* was only recorded on *M. natalensis* (Supplementary Tables 4 and 5). Five tick taxa (species and species groups) were recorded on *M. natalensis* and 4 on *M. coucha*. However, ticks were overall less prevalent on *M. natalensis* (6.93%) as opposed to *M. coucha* (22.79%). *Dermacentor rhinocerinus* was shared between the 2 rodents, while *Amblyomma hebraeum* and *Haemaphysalis zumpti* was only recorded on *M. natalensis* whereas *Hyalomma truncatum* was only recorded on *M. coucha* (Supplementary Tables 4 and 5). There were 19 mite species (excluding chiggers) recorded on *M. natalensis* and 12 recorded on *M. coucha*. Mites were less prevalent on *M. natalensis* (81.01%) compared to *M. coucha* (92.50%). Two parasitic mites *Laelaps liberiensis* and *L. muricola* were the most prevalent mite species on both rodent species (Supplementary Tables 4 and 5). Eight chigger species were recorded on *M. natalensis* and 6 on *M. coucha*. Chiggers were less prevalent on *M. natalensis* (25.32%) compared to *M. coucha* (45.00%). *Microtrombicula mastomyia* occurred on both rodent species, but at a lower prevalence on *M. natalensis* (22.36%) compared to *M. coucha* (36.67%) (Supplementary Table 6). On both rodent species several chiggers occurred on the ear pinna (Supplementary Table 6).

### *Mastomys natalensis* – Role of host- and habitat factors

**Figure 3.**
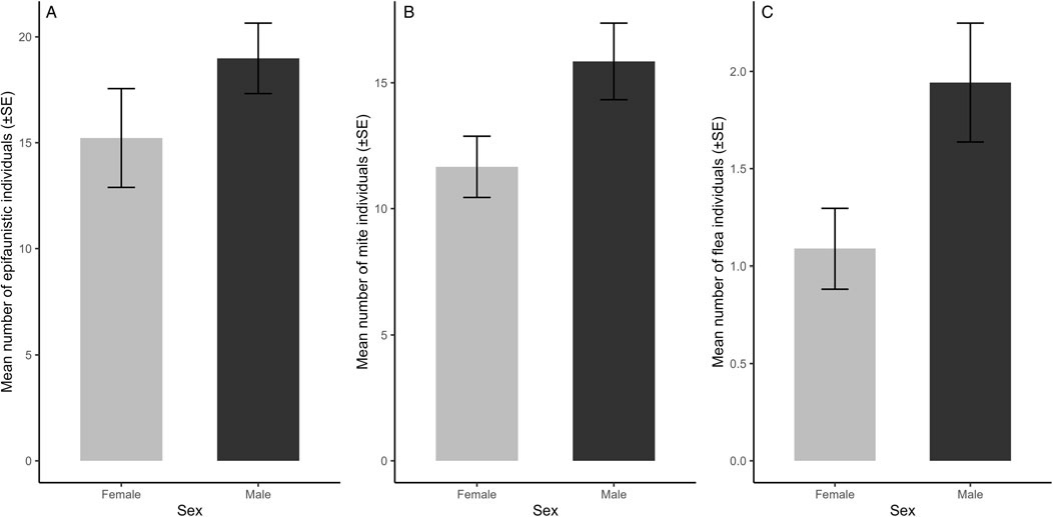
Mean number of: (a) epifaunistic, (b) mite and (c) flea individuals per host sex on *Mastomys natalensis* (*n* = 304) in Mpumalanga, South Africa (2014–2020).

**Figure 4.**
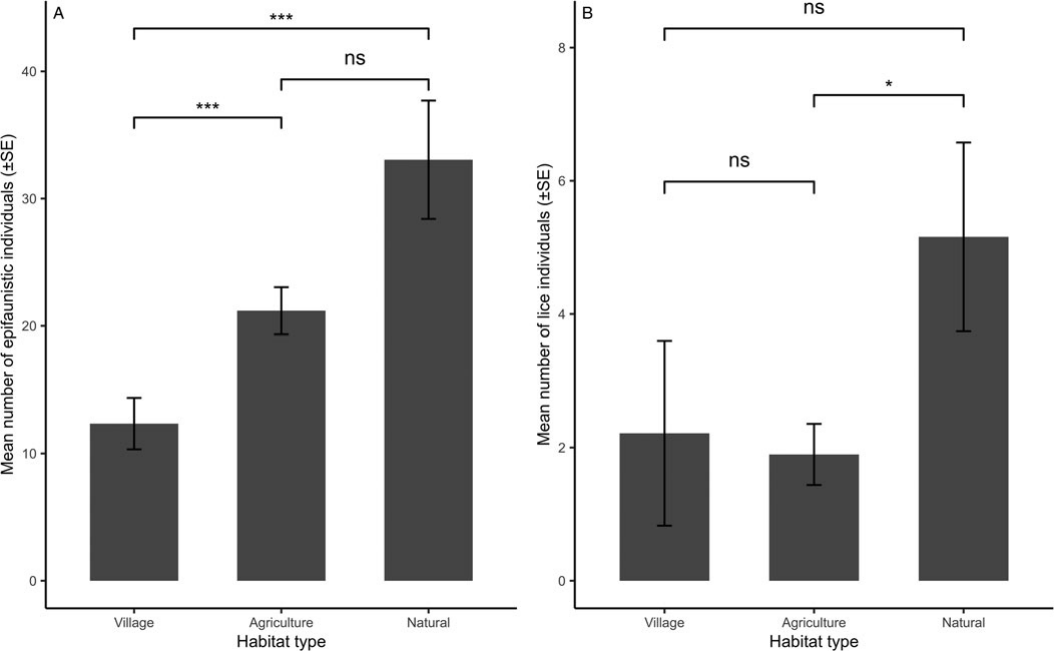
Mean number of: (a) epifaunistic and (b) lice individuals per habitat type on *Mastomys natalensis* (*n* = 304) in Mpumalanga, South Africa (2014–2020). Significant estimates: ***P < 0.001, **P < 0.01, *P < 0.05, ns-non-significant.

**Figure 5.**
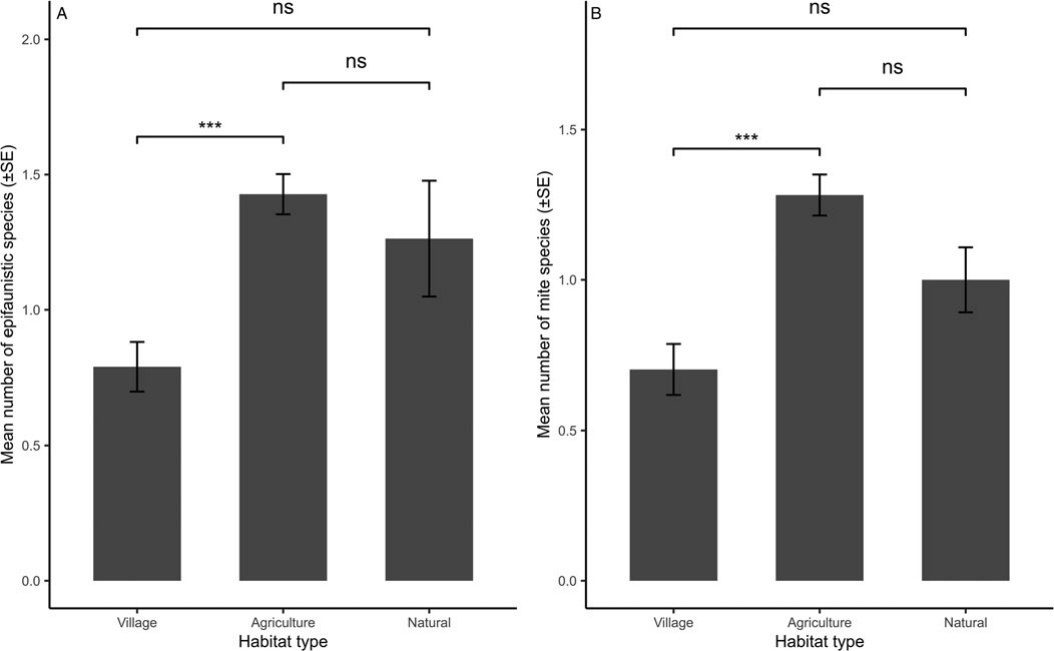
Mean number of: (a) epifaunistic, and (b) mite species per habitat type on *Mastomys natalensis* (*n* = 234) in Mpumalanga, South Africa (2014–2020). Significant estimates: ***P < 0.001, **P < 0.01, *P < 0.05, ns-non-significant.

The results of the final selected models are presented in Tables 1–3. Overall epifaunistic, mite and flea abundance were significantly related to host sex, with higher infestations on males compared to females (Tables 1 and 2; Fig. 3a–c). Tick abundance was significantly higher on breeding individuals, while louse occurrence was significantly higher on non-breeding rodents (Table 2). None of the infestation parameters (abundance or number of species) for overall epifauna, mites, fleas, lice and ticks on *M. natalensis* were significantly correlated with the interaction between sex and reproductive state and body size (Tables 1 and 2). In addition, none of the infestation parameters for overall epifauna, mites, fleas and ticks were significantly different between the agriculture and natural habitat types (Tables 1 and 2). However, overall epifaunistic, mite and tick abundances (Tables 1 and 2) were significantly lower in the village habitat type compared to the agricultural and natural habitat types (see Fig. 4a for epifaunistic abundance). Louse abundance was significantly higher on *M. natalensis* in the natural compared to the agricultural habitat type (Table 2; Fig. 4b). However, louse abundance did not differ between the agricultural and village habitat type, whereas louse occurrence was significantly higher on *M. natalensis* in the agricultural habitat compared to the village habitat type (Table 2). The number of epifauna, flea and mite species were significantly lower in the village compared to the agricultural habitat type (Table 3; Fig. 5a and b). Overall epifauna, tick abundance and the number of flea species were related to sampling year (Tables 1–3).

**Table 1.** Summary of the final selected generalized linear model with a Poisson distribution on the effect of host sex (SEX), reproductive state (BRS), year (Y) and habitat type (HBT) on the epifaunistic taxon abundance belonging to different higher taxa on *Mastomys natalensis* (*n* = 304, 2014–2020). Bold text indicate significant responses.

Taxon	Variable	Estimate	SE	*z*-value	*P* value
Overall epifaunistic individuals	BRS	0.284	0.165	1.721	0.085
	SEX	0.350	0.152	2.297	**0**.**022**
	HBT NAT: HBT AGR	0.180	0.146	1.230	0.219
	HBT VIL: HBT AGR	−0.466	0.106	−4.402	**<0.001**
	HBT NAT: HBT VIL	0.646	0.163	3.965	**<0.001**
	Y2015:Y2014	−0.069	0.178	−0.387	0.698
	Y2019:Y2014	−0.360	0.166	−2.163	**0**.**031**
	Y2020:Y2014	−0.307	0.169	−1.817	0.069
Mites	BRS	0.319	0.166	1.925	0.054
	SEX	0.324	0.158	2.052	**0**.**040**
	HBT NAT: HBT AGR	0.129	0.148	0.872	0.383
	HBT VIL: HBT AGR	−0.454	0.087	−5.199	**<0.001**
	HBT NAT: HBT VIL	0.583	0.156	3.742	**<0.001**
	BRS × SEX	−0.279	0.194	−1.440	0.150

Reference levels are male for SEX, non-breeding for BRS.

HBT NAT, natural habitat; HBT AGR, agricultural habitat; HBT VIL, village habitat.

**Table 2.** Summary of the final selected zero-inflation model with a Poisson distribution on the effect of host sex (SEX), reproductive state (BRS), tail length (TLL), year (Y) and habitat type (HBT) on the ectoparasite abundance belonging to different higher taxa on *Mastomys natalensis* (*n* = 304, 2014–2020). Bold text indicate significant responses.

Taxon	Variable	Estimate	SE	z-value	*P* value
Count model					
Fleas	SEX	0.471	0.179	2.641	**0**.**008**
	HBT NAT: HBT AGR	−0.422	0.330	−1.280	0.200
	HBT VIL: HBT AGR	−0.035	0.225	−0.153	0.878
	HBT NAT: HBT VIL	−0.388	0.369	−1.050	0.294
	Y2015:Y2014	−0.021	0.400	−0.053	0.957
	Y2019:Y2014	0.191	0.421	0.454	0.650
	Y2020:Y2014	−0.281	0.407	−0.691	0.490
Lice	BRS	−0.460	0.239	−1.922	0.055
	HBT NAT: HBT AGR	0.655	0.272	2.405	**0**.**016**
	HBT VIL: HBT AGR	0.392	0.265	1.479	0.139
	HBT NAT: HBT VIL	0.263	0.324	0.811	0.417
Ticks	TLL	0.412	0.356	1.158	0.247
	BRS	−3.161	1.117	−2.830	**0**.**005**
	HBT NAT: HBT AGR	0.973	1.198	0.812	0.417
	HBT VIL: HBT AGR	−3.039	1.074	−2.828	**0**.**005**
	HBT NAT: HBT VIL	5.316	1.108	4.798	**<0.001**
	Y2015:Y2014	−4.738	1.773	−2.672	**0**.**008**
	Y2019:Y2014	−2.267	1.251	−1.813	0.070
	Y2020:Y2014	−5.218	1.330	−3.923	**<0.001**
Zero model					
Fleas	SEX	1.498	1.322	1.133	0.257
	HBT NAT: HBT AGR	−0.569	1.156E3	0.000	1.000
	HBT VIL: HBT AGR	14.370	323.900	0.044	0.965
	HBT NAT: HBT VIL	−12.427	316.804	−0.039	0.969
	Y2015:Y2014	−0.298	1.53E6	0.000	1.000
	Y2019:Y2014	0.931	1.042	0.893	0.372
	Y2020:Y2014	−1.069	1.640	−0.651	0.515
Lice	BRS	−3.111	0.936	−3.323	**<0.001**
	HBT NAT: HBT AGR	−2.674	3.386	−0.494	0.621
	HBT VIL: HBT AGR	2.539	0.687	3.698	**<0.001**
	HBT NAT: HBT VIL	−4.212	3.397	−1.240	0.215
Ticks	TLL	0.388	0.590	0.657	0.511
	BRS	−20.433	188.366	−0.108	0.914
	HBT NAT: HBT AGR	11.154	67.298	−0.166	0.868
	HBT VIL: HBT AGR	29.468	129.561	−0.134	0.893
	HBT NAT: HBT VIL	15.999	93.113	−0.172	0.864
	Y2015:Y2014	43.901	212.537	−0.207	0.836
	Y2019:Y2014	−5.775	93.943	−0.061	0.951
	Y2020:Y2014	26.104	214.986	−0.121	0.903

Reference levels are male for SEX, non-breeding for BRS.

HBT NAT, natural habitat; HBT AGR, agricultural habitat; HBT VIL, village habitat.

**Table 3.** Summary of the final selected generalized liner model with a Poisson distribution on the effect of host reproductive state (BRS), tail length (TLL), year (Y) and habitat type (HBT) on number of epifaunistic, flea and mite species on *Mastomys natalensis* (*n* = 234, 2019–2020). Bold text indicate significant responses.

Taxon	Variable	Estimate	SE	*z*-value	*P* value
Epifaunistic taxa	TLL	−0.088	0.063	−1.410	0.158
	HBT NAT: HBT AGR	−0.116	0.218	−0.534	0.593
	HBT VIL: HBT AGR	−0.558	0.142	−3.938	**<0.001**
	HBT NAT: HBT VIL	0.442	0.237	1.867	0.062
Fleas	HBT NAT: HBT AGR	−1.522	1.046	−1.455	0.146
	HBT VIL: HBT AGR	−1.120	0.482	−2.323	**0**.**020**
	HBT NAT: HBT VIL	−0.402	1.061	−0.379	0.705
	Y2020	−1.265	0.442	−2.860	**0**.**004**
Mites	HBT NAT: HBT AGR	−0.249	0.243	−1.024	0.306
	HBT VIL: HBT AGR	−0.601	0.148	−4.057	**<0.001**
	HBT NAT: HBT VIL	0.352	0.261	1.347	0.178

Reference levels are male for SEX, non-breeding for BRS, 2019 for YEAR.

HBT NAT, natural habitat; HBT AGR, agricultural habitat; HBT VIL, village habitat.

### *Mastomys coucha* – Role of host- and habitat factors

**Figure 6.**
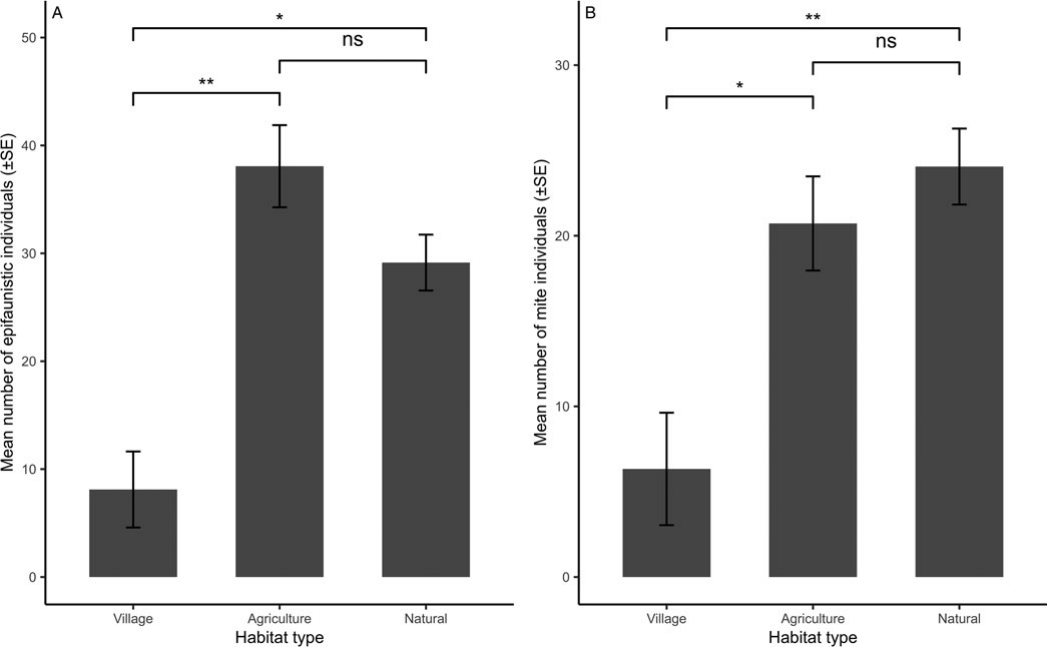
Mean number of: (a) epifaunistic, and (b) mite individuals per habitat type for *Mastomys coucha* in Mpumalanga, South Africa (2014–2020). Significant estimates: ***P < 0.001, **P < 0.01, *P < 0.05, ns-non-significant.

The results of the final selected models are presented in Tables 4–6. Louse occurrence was significantly negatively related to host body size (i.e. higher occurrence on smaller-bodied rodents; Table 5). None of the infestation parameters for overall epifauna, mites, fleas and ticks on *M. coucha* were significantly associated with host sex, reproductive state, the interaction between sex and reproductive state and body size (Tables 4 and 5). Additionally, none of the infestation parameters for overall epifauna, mites, fleas and ticks were significantly different between the agriculture and natural habitat types. However, overall epifauna and mite abundance were significantly lower in the village habitat type compared to the agricultural and the natural habitat type (Table 4; Fig. 6a and b). Further, louse occurrence was significantly higher on *M. coucha* in the agricultural habitat type compared to the natural and village habitat type and higher in the natural habitat type when compared to the village habitat type (Table 5). Flea and louse abundance, and flea species were significantly related to sampling year (Tables 5 and 6).

**Table 5.** Summary of the final selected zero-inflated model (count and zero model) with a Poisson distribution on the effect of host sex (SEX), reproductive state (BRS), tail length (TLL), year (Y) and habitat type (HBT) on the ectoparasite abundance belonging to different higher taxa on *Mastomys coucha* (*n* = 189, 2014–2020). Bold text indicate significant responses.

Taxon	Variable	Estimate	SE	z-value	*P* value
Count model					
Fleas	TLL	0.221	0.190	1.162	0.245
	BRS	0.474	0.414	1.143	0.253
	SEX	−0.350	0.265	−1.324	0.185
	Y2015:Y2014	1.561	0.455	3.435	**<0.001**
	Y2019:Y2014	1.605	0.429	3.740	**<0.001**
	Y2020:Y2014	0.749	0.531	1.410	0.158
Lice	TLL	0.058	0.107	0.547	0.585
	HBT NAT: HBT AGR	0.140	0.184	0.758	0.448
	HBT VIL: HBT AGR	0.355	0.865	0.410	0.682
	HBT NAT: HBT VIL	−0.215	0.857	−0.251	0.802
	Y2015:Y2014	−1.774	0.418	−4.249	**<0.001**
	Y2019:Y2014	−0.947	0.255	−3.687	**<0.001**
	Y2020:Y2014	−1.397	0.244	−5.721	**<0.001**
Ticks	TLL	0.118	0.243	0.486	0.627
	SEX	−0.524	0.361	−1.452	0.147
Zero model					
Fleas	TLL	−1.072	1.905	−0.563	0.574
	BRS	2.169	8.739	0.248	0.804
	SEX	−3.987	20.081	−0.199	0.843
	Y2015:Y2014	−10.796	204.626	−0.053	0.958
	Y2019:Y2014	−17.042	7805.506	−0.002	0.998
	Y2020:Y2014	1.859	3.478	0.535	0.593
Lice	TLL	−1.934	0.803	−2.409	**0**.**016**
	HBT NAT: HBT AGR	2.448	1.010	2.424	**0**.**015**
	HBT VIL: HBT AGR	6.961	2.434	2.859	**0**.**004**
	HBT NAT: HBT VIL	−4.513	1.970	−2.291	**0**.**022**
	Y2015:Y2014	−1.827	2.489	−0.734	0.463
	Y2019:Y2014	−1.904	1.325	−1.437	0.151
	Y2020:Y2014	−1.585	1.157	−1.370	0.171
Ticks	TLL	8.242	17.018	0.484	0.628
	SEX	15.862	29.328	0.541	0.589

Reference levels are male for SEX, non-breeding for BRS.

HBT NAT, natural habitat; HBT AGR, agricultural habitat; HBT VIL, village habitat.

**Table 4.** Summary of the final selected generalized linear model with a Poisson distribution on the effect of habitat type (HBT) on the epifaunistic taxon abundance belonging to different higher taxa on *Mastomys coucha* (*n* = 189, 2014–2020). Bold text indicate significant responses.

Taxon	Variable	Estimate	SE	*z*-value	*P* value
Overall epifaunistic individuals	HBT NAT: HBT AGR	−0.132	0.087	−1.511	0.131
	HBT VIL: HBT AGR	−0.820	0.285	−2.517	**0**.**004**
	HBT NAT: HBT VIL	0.688	0.283	2.375	**0**.**015**
Mites	HBT NAT: HBT AGR	0.030	0.094	0.314	0.754
	HBT VIL: HBT AGR	−0.762	0.311	−2.451	**0**.**014**
	HBT NAT: HBT VIL	0.791	0.307	2.578	**0**.**010**

HBT NAT, natural habitat; HBT AGR, agricultural habitat; HBT VIL, village habitat.

**Table 6.** Summary of the final selected generalized linear model with a Poisson distribution on the effect of host sex (SEX), year (Y) and habitat type (HBT) on number of epifaunistic, flea and mite species on *Mastomys coucha* (*n* = 118, 2019–2020). Bold text indicate significant responses.

Taxon	Variable	Estimate	SE	*z*-value	*P* value
Epifaunistic species	HBT NAT: HBT AGR	−0.204	0.227	−0.899	0.369
	HBT VIL: HBT AGR	−0.981	0.540	−1.816	0.069
	HBT NAT: HBT VIL	0.777	0.510	−1.816	0.128
Fleas	Y2020	−3.273	1.054	−3.106	**0.002**
Mites	HBT NAT: HBT AGR	−0.116	0.247	−0.471	0.638
	HBT VIL: HBT AGR	−0.109	0.619	−1.754	0.079
	HBT NAT: HBT VIL	0.970	0.587	1.653	0.098

Reference levels are male for SEX, 2019 for YEAR.

HBT NAT, natural habitat; HBT AGR, agricultural habitat; HBT VIL, village habitat.

## Discussion

### Epifaunistic diversity

The 2 rodent species only shared approximately 50% of the epifaunistic species. This is most probably the result of the observed variation in spatial occurrence between the rodents with *M. natalensis* occurring mainly in the agricultural and village habitats, while *M. coucha* occurred mainly in the agricultural and natural habitats (Stenseth *et al*., 2001; Garba *et al*., 2014; McCauley *et al*., 2015). Habitat-type associated variance was also recorded in other co-occurring rodent species in the present study. In particular, *Rattus rattus* and *Rattus tanezumi* were trapped predominantly in the village habitat, while *Gerbilliscus leucogaster*, *Saccostomus campestris* and *Lemniscomys rosalia* overlapped with *M. coucha* (Matthee *et al*., 2020; Smith *et al*., 2023). Based on this it is evident that the village habitat harboured fewer rodent species compared to the agricultural and natural habitats. The parasite diversity on *M. natalensis* and *M. coucha* may thus be directly related to variation in abundance and diversity of rodent hosts and their parasites in the different habitat types (also see Krasnov *et al*., 2004; Morand *et al*., 2006). Similarly, the variation in relative density across habitat types for both *Mastomys* species will influence their contact with conspecific and confamilial host species and ectoparasites in the environment (Cote and Poulin, 1995; Arneberg *et al*., 1998). This is consistent with previous studies that recorded a positive relationship between host density and parasite infestations (Esch and Fernández, 1993; Tompkins *et al*., 2001; Rifkin *et al*., 2012). It is thus most likely that habitat type-related host diversity and relative abundance contributed to the fact that only 50% of epifaunistic species were shared between the 2 rodents (Haukisalmi *et al*., 1987; Froeschke and Matthee, 2014).

The occurrence of *X. brasiliensis* on *M. natalensis* is well documented (Isaacson, 1975; Segerman, 1995; Guerra *et al*., 2016) and in fact *M. natalensis* together with *Rattus* spp. (also commensal) are the principal hosts for the flea (Segerman, 1995). The present study provides the first record of *X. brasiliensis* on *M. coucha* in South Africa. This flea is a known vector of several zoonotic pathogens such as the bacterium *Y. pestis* and *Bartonella* species in Africa (Zimba *et al*., 2011; Billeter *et al*., 2014). In the study, higher infestations of *X. frayi* were recorded on *M. coucha* compared to *M. natalensis*. This may be due to the fact that *G. leucogaster* is the principal host of *X. frayi* (Segerman, 1995) and the rodent co-occurred with *M. coucha* in the agricultural and natural habitat type (Smith *et al*., 2023). Given that *M. natalensis,* and most likely *M. coucha,* often use the abandoned burrows of other rodent species (Veenstra, 1958; Coetzee, 1975; Isaacson, 1975) the occurrence of non-specific flea species on *Mastomys* is most likely facilitated through nest sharing.

Current taxonomic records list the louse *H. intermedia* on *M. natalensis* and *M. coucha* (Ledger, 1980; Durden and Musser, 1994). Important to realize, however, co-evolutionary divergence among lice and their hosts are often found (du Toit *et al*., 2013; Bothma *et al*., 2020, 2021; Durden *et al*., 2020) and it is thus quite possible that *H. intermedia* comprises of 2 cryptic species. The presence of *P. biseriata* on *M. coucha* is possibly an accidental infestation, given that *G. leucogaster* is the principal host of this taxon (Ledger, 1980; Durden and Musser, 1994). Similarly, the presence of *P. spinulosa* on *M. natalensis* may also be accidental given that the principal host is *R. rattus* (Durden and Musser, 1994). Studies have recorded that *M. natalensis* (and possibly *M. coucha*) are rarely aggressive to conspecifics and/or other rodent species (Veenstra, 1958) and accidental parasite exchange is quite plausible.

Overall ticks were the least prevalent taxon on both rodent species studied herein. Many ixodid tick species, and especially the taxa recorded in the present study, require vegetation (e.g. grass) to attach to and wait for a host to pass (Horak *et al*., 2018; Ledger *et al*., 2019). In the present study, the village habitat lacked natural vegetation and had proportionally lower cover compared to the agricultural and natural habitat type (Matthee *et al*., 2020; S. Matthee personal observation). This may contribute to the overall lower tick infestations on *M. natalensis*. The larvae and or nymphs of 3 of the 4 species that could be identified (*A. hebraeum, D. rhinocerinus,* and *H. truncatum*) are known to occur on murid rodents (Horak *et al*., 2018). However, the single occurrence of *Hae. zumpti* (1 individual) is most probably related to the fact that the tick prefers Sciuridae and Carnivora (Horak *et al*., 2018; Jongejan *et al*., 2020).

Mites (excluding chiggers) were the most specious taxon with *L. liberiensis* and *L. muricola* (both parasitic species) being the most common on both rodents. *Mastomys natalensis* is a new host record for *Androlaelaps oliffi, A. rhodesiensis, A. taterae* and *L. simillimus*. The presence of the remaining *Androlaelaps* and *Laelaps* species on *M. natalensis* is supported by previous taxonomic records (Isaacson, 1975). Apart from *L. muricola* (previously recorded on *M. coucha*, Engelbrecht *et al*., 2014) the remaining *Androlaelaps* and *Laelaps* species are new records for *M. coucha*. The presence of *Ornithonyssus bacoti,* known as the tropical rat mite, represents the first record of this mite species on *M. natalensis*. The mite was recorded on *M. natalensis* in the village and agricultural habitat types but was absent from conspecific individuals trapped in the natural habitat. The primary hosts of *O. bacoti* are *R. tanezumi* and *R. rattus*, and as mentioned before both rodent species co-occurred with *M. natalensis* in the village habitat. This mite can cause pruritic papular dermatitis (known as rat mite dermatitis) in humans (Feldman and Easton, 2005; Clancy *et al*., 2022).

Chiggers were less prevalent on *M. natalensis* (25.32%) as opposed to *M. coucha* (45%). Six chigger species were shared between the 2 *Mastomys* species. Chiggers are regarded as habitat specialists and can occur on multiple unrelated host species in a particular habitat (Sasa, 1960). The present study provides the first list of chigger species recorded for *M. coucha* in South Africa. The ear pinna was the preferred attachment site for chiggers on both rodents. This parasitope was also recorded for several other rodents in South Africa (Fagir *et al*., 2014; Matthee *et al*., 2020; Stevens *et al*., 2022). It is possible that this attachment site is preferred due to the protection that it provides against parasite removal through grooming.

### Role of host- and habitat-associated factors on the epifauna of *M. natalensis* and *M. coucha*

Male-biased parasitism was recorded for overall epifauna, mite and flea abundance on *M. natalensis*. This pattern supports previous studies on other rodent species in South Africa (Archer *et al*., 2014; Smith *et al*., 2023) and elsewhere (Krasnov *et al*., 2005). There are several, not mutually exclusive, factors that can contribute to this pattern. In particular, large-bodied male hosts encounter and can accommodate more ectoparasites due to their larger surface area and available niche space for parasites (Krasnov *et al*., 1997; Ezenwa *et al*., 2006), males are less likely to engage in grooming activities compared to females (Ferkin and Leonard, 2007; Hawlena *et al*., 2007) and males of some rodent species increase their home range during the breeding season (in search for females) which increases their contact with ectoparasites (i.e. mites, fleas, ticks) (Perez-orella and Schulte-hostedde, 2005; Morand *et al*., 2006; Frafjord, 2016). Linked to this, during the breeding season elevated testosterone levels can facilitate immune suppression that may further increase their susceptibility to parasites (Klein, 2004; Stanko *et al*., 2015). In the present study, male *M. natalensis* were larger (9.61 cm ± 0.05) compared to females (9.08 cm ± 0.06)) and more than 50% of the individuals were in the breeding state. Furthermore, from previous studies (Mlyashimbi *et al*., 2019; Goyens *et al*., 2020) male *M. natalensis* increase their home range during the breeding season, thereby increasing their exposure to free-living infective life stages in the environment. The above mentioned may also explain why tick counts were significantly higher on *M. natalensis* individuals that were in the breeding state compared to non-breeding individuals. In contrast to the tick pattern, lice were significantly more prevalent on non-breeding *M. natalensis* individuals compared to breeding individuals. Lice are transmitted through direct body contact and it is possible that non-breeding *M. natalensis* spend more time engaging with conspecifics in the nest (Choate, 1972).

From the study it seems that host-related parameters were less important in shaping epifaunistic infestations on *M. coucha*. It is possible that the fact that a higher proportion of *M. coucha* individuals were in the non-breeding state (83.07%) compared to the breeding individuals (16.93%) contributed to this. However, louse prevalence was significantly higher on smaller-bodied *M. coucha* compared to larger individuals. This pattern is in agreement with the pattern recorded for *M. natalensis* (higher louse prevalence on non-breeding individuals).

Epifaunistic abundance and particularly overall, flea, tick and mite abundance, on *M. natalensis* and *M. coucha* were not significantly different between the agricultural and natural habitat types. In contrast, the abundance of epifaunistic individuals, mites and ticks (the latter for *M. natalensis*) were significantly lower in the village compared to the agricultural and natural habitats. In addition, epifaunistic, flea and mite species richness on *M. natalensis* were also significantly lower in the village compared the agricultural habitat type. These patterns may be due to higher similarity in the vegetation structure (Matthee *et al*., 2020) and rodent species in the agricultural and natural habitats compared to the village. Another contributing factor may be the fact that ectoparasite infestations were very low on the 2 *Rattus* species that co-occurred in the village habitat with *M. natalensis* (unpublished data). Depauperate parasite communities associated with *Rattus* species have also been recorded elsewhere (Murrel and Cates, 1970; Alonso *et al*., 2020). Lower rodent diversity in addition in a poorer parasite community most probably contributes to the lower ectoparasite abundances and species richness in the village. It is interesting to note that lice were significantly more abundant on *M. natalensis* in the natural compared to the agricultural habitat type but was not significantly different from the village habitat when compared to the agricultural and natural habitat type. Although fewer *M. natalensis* individuals were trapped in the natural habitat (19) the total louse abundance was 98 individuals and 6 *M. natalensis* individuals harboured >10 louse individuals. Whereas the 136 *M. natalensis* individuals in the agricultural habitat harboured a total of 258 lice and only 8 individuals carried >10 louse individuals. Therefore, a higher proportion of *M. natalensis* had higher louse counts in the natural habitat type (31.57%) when compared to the agricultural habitat (5.88%). However, lice were more prevalent on *M. natalensis* and *M. coucha* in the agricultural habitat type compared to the village habitat. Higher host density can facilitate contact between conspecifics which may result in louse transfer (Arneberg *et al*., 1998). However, in the present study, the densities of *M. natalensis* was the highest in the village (66.88%) followed by the agriculture habitat type (43.44%), while *M*. *coucha* had the highest density recorded in the natural (37.81%) followed by the agriculture habitat type (25.94%). Regarding *M*. *natalensis*, the agricultural habitat type had a higher proportion of non-breeding individuals (53.70%) when compared to the village (36.90%), fitting the pattern that lice are more prevalent on non-breeding *M. natalensis* individuals compared to breeding individuals. In addition, Borremans *et al*. (2016) found that at high host densities *M. natalensis* spend less time in contact with each other, whereas at a lower host density they spend more time together. Similar to *M. natalensis*, a higher proportion of non-breeding compared to breeding individuals were recorded for *M. coucha* in the agriculture (86.80%) compared to village habitat (55.50%). This pattern is also in agreement with the abovementioned record that lice were significantly more prevalent on smaller bodied (and most possibly non-breeding) *M. coucha*. However, lice prevalence was also significantly higher on *M. coucha* in the agricultural habitat type compared to the natural habitat type. It is uncertain what drives this pattern as the proportion of non-breeding compared to breeding individuals and their respective body sizes were not distinctly different between the agricultural and natural habitat types. It is possible that host density played a role as the relative density of *M. coucha* was higher in the natural compared to the agricultural habitat type. It is however important to note that previous studies on rodents have suggested that high host density does not always indicate a higher rate of contact between rodents (Froeschke *et al*., 2013; Stanko *et al*., 2015), which also agrees with the study by Borremans *et al*. (2016).

Recording the between-sampling year variation in infestations was not the focus of the study, however, given that epifaunistic species are ectothermic and sensitive to climatic conditions (Altizer *et al*., 2006; Paaijmans *et al*., 2013) it is expected that infestations will be influenced by annual variation in climatic conditions, a scenario that is open for further investigation in the future.

This study represents the first systematic long-term assessment of the ectoparasite species associated with *M. natalensis* and *M. coucha*. The findings support previous studies in that widely distributed rodent species harbour a diverse set of parasite species. Evident from the study is that habitat preference and the diversity of the local rodent community play important determining roles in shaping ectoparasite infestations, even in closely related host species such as *M. natalensis* and *M. coucha*.

## Supporting information

Little et al. supplementary material Little et al. supplementary material

## Data Availability

All data generated or analysed during this study are included in this published article. The datasets used and/or analysed are available from the corresponding author upon reasonable request.

## Supplementary material

The supplementary material for this article can be found at https://doi.org/10.1017/S0031182024000714.
