# Supplementary material for: Host and habitat shape ectoparasite diversity on Mastomys natalensis and Mastomys coucha (Muridae)
Source: Parasitology. 2024 Oct 23;151(8):769–83. doi: 10.1017/S0031182024000714 (PMC11579041; doi:10.1017/S0031182024000714)
Supplement: Little et al. supplementary material [file S0031182024000714sup001.docx]

**Supplementary Material**

**Supplementary Table 1**. Sampling period and sample size for *Mastomys natalensis* and *Mastomys coucha* trapped across a wildlife-human/domestic animal interface in Mpumalanga, South Africa (2014-2020).

| Collection date | *Mastomys natalensis* | *Mastomys coucha* |
| --- | --- | --- |
| August 2014 | 70 | 74 |
| January 2015 | 28 | 4 |
| September 2015 | 40 | 17 |
| September 2019 | 70 | 32 |
| October 2020 | 167 | 88 |
| **Total** | **375** | **215** |

**Supplementary Table 2**: Relative host density for *Mastomys natalensis* (n = 375) and *Mastomys coucha* (n = 215) in different habitat types across a wildlife-human/domestic animal interface in Mpumalanga, South Africa (2014-2020).

|  | Relative host density (%) | |
| --- | --- | --- |
| Habitat type | *Mastomys natalensis* | *Mastomys coucha* |
| Village | 66.88 | 3.44 |
| Agriculture | 43.44 | 25.94 |
| Natural | 6.88 | 37.81 |

**Supplementary Table 3.** Epifaunistic species recorded on *Mastomys natalensis* (n = 375) and *Mastomys coucha* (n = 215) across a wildlife-human/domestic animal interface in Mpumalanga, South Africa, 2014-2020.

|  | **Order** | **Suborder** | **Family/Subfamily** | **Species** | ***M. natalensis*** | ***M. coucha*** |
| --- | --- | --- | --- | --- | --- | --- |
| **Fleas** | Siphonaptera |  | **Hystrichopsyllidae** |  |  |  |
|  |  |  | Listropsyllinae | *Listropsylla prominens* Jordan, 1930 | - | x |
|  |  |  | **Pulicidae** |  |  |  |
|  |  |  | Archaeopsyllinae | *Ctenocephalides felis* (Bouché, 1835) | x | - |
|  |  |  | Pulicinae | *Echidnophaga gallinacea* (Westwood, 1875) | x | x |
|  |  |  | Xenopsyllinae | *Pariodontis* sp. | - | x |
|  |  |  |  | *Xenopsylla bechuanae* De Meillon, 1947 | x | x |
|  |  |  |  | *Xenopsylla brasiliensis* (Baker, 1904) | x | x |
|  |  |  |  | *Xenopsylla frayi* De Meillon, 1947 | x | x |
| **Lice** | Phthiraptera | Anoplura | **Hoplopleuridae** | *Hoplopleura intermedia* Kellogg & Ferris, 1915 | x | x |
|  |  |  |  | *Hoplopleura intermedia/ismailiae* | x | *-* |
|  |  |  | **Polyplacidae** | *Polyplax biseriata* Ferris, 1923 | - | x |
|  |  |  |  | *Polyplax spinulosa* (Burmeister, 1839) | x | - |
| **Ticks** | Ixodida |  | **Ixodidae** |  |  |  |
|  |  |  | Amblyomminae | *Amblyomma hebraeum* Koch, 1844 | x | - |
|  |  |  |  | *Dermacentor rhinocerinus* (Denny, 1843) | x | x |
|  |  |  | Haemaphysalinae | *Haemaphysalis leachi/elliptica* group | x | x |
|  |  |  |  | *Haemaphysalis zumpti* Hoogstraal and El Kammah, 1974 | x | - |
|  |  |  | Hyalomminae | *Hyalomma truncatum* Koch, 1844 | - | x |
|  |  |  | Rhipicephalinae | *Rhipicephalus follis/simus*group | x | x |
| **Mites** | Mesostigmata | Monogynaspida | **Ameroseiidae** | *Ameroseiidae* sp. | - | x |
|  |  |  | **Laelapidae** |  |  |  |
|  |  |  | Laelaptinae | *Androlaelaps* sp. | x | - |
|  |  |  |  | *Androlaelaps marshalli* Berlese, 1911 | x | x |
|  |  |  |  | *Androlaelaps oliffi* (Zumpt & Patterson, 1951) | x | x |
|  |  |  |  | *Androlaelaps rhabdomysi* Matthee & Ueckermann, 2008 | - | x |
|  |  |  |  | *Androlaelaps rhodesiensis* (Zumpt & Patterson, 1951) | x | - |
|  |  |  |  | *Androlaelaps taterae* (Zumpt & Patterson, 1951) | x | - |
|  |  |  |  | *Androlaelaps theseus* Zumpt, 1950 | x | - |
|  |  |  |  | *Androlaelaps zuluensis* Zumpt, 1950 | - | x |
|  |  |  |  | *Laelaps giganteus* Berlese, 1918 | x | x |
|  |  |  |  | *Laelaps liberiensis* Hirst, 1925 | x | x |
|  |  |  |  | *Laelaps muricola* Trägårdh, 1910 | x | x |
|  |  |  |  | *Laelaps vansomereni* Hirst, 1923 | x | *-* |
|  |  |  |  | *Laelaps simillimus* Zumpt, 1950 | x | - |
|  |  |  |  | *Laelaps tillae* Taufflieb, 1959 | x | - |
|  |  |  | **Macronyssidae** | Macronyssidae sp. | x | - |
|  |  |  |  | *Ornithonyssus bacoti* (Hirst, 1913) | x | - |
|  |  |  | **Pachylaelapidae** | *Pachylaelaps* sp. ^p^ | x | x |
|  |  |  | **Uropodoidae** | *Uropodoidae* sp. ^p^ | x | x |
|  | Sarcoptiformes | Oribatida | **Atopmelidae** | *Listrophoroides (Afrolistrophoroides) mastomys* Radford, 1940 | x | x |
|  |  |  | **Listrophoridae** | Listrophoridae sp. | x | - |
|  | Trombidiformes | Prostigmata | **Cheyletidae** | *Cheyletus zumpti* Fain, 1972^p^ | x | x |
|  |  |  | **Myobiidae** | *Austromyobia forcipifer* Lawrence, 1954 | - | x |
|  |  |  | **Trombiculidae** |  |  |  |
|  |  |  | Gahrliepiinae | *Gahrliepia nana* (Oudemans, 1910) | x | x |
|  |  |  | Trombiculinae | *Ascoschoengastia ueckermanni* Stekolnikov & Matthee, 2019 | x | x |
|  |  |  |  | *Hypotrombidium clamatori* (Vercammen-Grandjean and Langston, 1976) | x | x |
|  |  |  |  | *Susa hexasternalaea* (Vercammen-Grandjean, 1960) | x | - |
|  |  |  |  | *Microtrombicula mastomyia* (Radford, 1942) | x | x |
|  |  |  |  | *Schoutedenichia dutoiti* (Radford, 1948) | x | x |
|  |  |  |  | *Schoutedenichia lumsdeni* Vercammen-Grandjean, 1958 | x | x |
|  |  |  |  | *Schoutedenichia morosi* Vercammen-Grandjean, 1958 | x | - |

^p^Predatory feeding, x epifaunistic species present. - epifaunistic species absent.

**Supplementary Table 4.** Life stages and infestation parameters of epifaunistic species recorded on *Mastomys natalensis* (n = 375) across a wildlife-human/domestic animal interface in Mpumalanga, South Africa, 2014-2020.

| Taxa | Total no. of individuals | Adults (%) | Larvae (%) | Nymphs (%) | Adult Male (%) | Adult Female (%) | Mean Abundance (±SE) | Prevalence (%) |
| --- | --- | --- | --- | --- | --- | --- | --- | --- |
| **Fleas*** | **586** | **100** | - | - | **42.83** | **57.17** | **1.56 (±0.15)** | **46.93** |
| *Ctenocephalides felis*** | 1 | 100 | - | - | - | - | - | 0.27 |
| *Echidnophaga gallinacea*** | 9 | 100 | - | - | - | - | 0.02 (±0.01) | 1.07 |
| *Xenopsylla bechuanae*** | 5 | 100 | - | - | - | - | 0.01 (±0.01) | 1.33 |
| *X. brasiliensis*** | 210 | 100 | - | - | - | - | 0.56 (±0.07) | 27.20 |
| *X. frayi*** | 25 | 100 | - | - | - | - | 0.07 (±0.02) | 4.53 |
| **Lice** | **914** | **70.57** | - | **29.43** | **35.35** | **64.65** | **2.44 (±0.61)** | **33.33** |
| *Hoplopleura intermedia* | 890 | 70 | - | 30 | 35.63 | 64.37 | 2.37 (±0.57) | 32.53 |
| *Hoplopleura intermedia/ismailiae* | 18 | 100 | - | 0 | 11.11 | 88.89 | 0.05 (±0.04) | 0.80 |
| *Polyplax spinulosa* | 6 | 66.67 | - | 33.33 | 100 | 0 | 0.02 (±0.01) | 0.80 |
| **Ticks** | **79** | **-** | **72.15** | **27.85** | **-** | **-** | **0.21 (±0.07)** | **6.93** |
| *Amblyomma hebraeum* | 7 | - | 57.14 | 42.86 | - | - | 0.02 (±0.01) | 1.33 |
| *Dermacentor rhinocerinus* | 5 | - | 40.00 | 60.00 | - | - | 0.01 (±0.01) | 0.27 |
| *Haemaphysalis leachi/elliptica* group | 61 | - | 81.96 | 18.03 | - | - | 0.16 (±0.06) | 5.07 |
| *Hae. zumpti* | 1 | - | 100 | 0 | - | - | - | 0.27 |
| *Rhipicephalus follis/simus*group | 5 | - | 0 | 100 | - | - | 0.01 (±0.01) | 0.80 |
| **Mites** *^#^* | **3474** | **76.54** | **0.09** | **23.37** | **29.52** | **70.48** | **9.26 (±1.03)** | **81.01** |
| *Androlaelaps* sp. | 5 | 100 | 0 | 0 | 0 | 100 | 0.01 (±0.01) | 0.42 |
| *A. marshalli* | 24 | 88.33 | 0 | 16.67 | 25 | 75 | 0.06 (±0.03) | 6.33 |
| *A. oliffi* | 1 | 100 | 0 | 0 | 100 | 0 | - | 0.42 |
| *A. rhodesiensis* | 1 | 100 | 0 | 0 | 0 | 100 | - | 0.42 |
| *A. taterae* | 1 | 100 | 0 | 0 | 0 | 100 | - | 0.42 |
| *A. theseus* | 1 | 100 | 0 | 0 | 0 | 100 | - | 0.42 |
| *Cheyletus zumpti* ^p^ | 3 | 100 | 0 | 0 | 0 | 100 | 0.01 (±0.01) | 1.27 |
| *Laelaps giganteus* | 2 | 100 | 0 | 0 | 0 | 100 | 0.01 (±0) | 0.84 |
| *L. liberiensis* | 3002 | 74.88 | 0.10 | 25.02 | 33.10 | 66.90 | 8.01 (±0.95) | 75.11 |
| *L. muricola* | 319 | 99.69 | 0 | 0.31 | 4.09 | 95.91 | 0.85 (±0.11) | 48.95 |
| *L. simillimus* | 12 | 91.67 | 0 | 8.33 | 18.18 | 81.82 | 0.03 (±0.04) | 0.42 |
| *L. tillae* | 76 | 39.47 | 0 | 60.53 | 63.33 | 36.67 | 0.20 (±0.25) | 0.42 |
| *L. vansomereni* | 3 | 100 | 0 | 0 | 0 | 100 | 0.01 (±0.01) | 0.84 |
| Listrophoridae sp. | 10 | 100 | 0 | 0 | 0 | 100 | 0.03 (±0.02) | 2.11 |
| *Listriohoroides (A.) mastomys* | 3 | 100 | 0 | 0 | 0 | 100 | 0.01 (±0.01) | 0.84 |
| Macronyssidae sp. | 1 | 100 | 0 | 0 | 100 | 0 | - | 0.42 |
| *Ornithonyssus bacoti* | 4 | 0 | 0 | 100 | 0 | 0 | 0.01 (±0.01) | 0.84 |
| *Pachylaelaps*sp. ^p^ | 1 | 100 | 0 | 0 | 0 | 100 | - | 0.42 |
| Uropodoidae sp. ^p^ | 5 | 0 | 0 | 100 | 0 | 0 | 0.01 (±0.02) | 0.42 |
| **Chiggers***^#^* | - | - | - | - | - | - | - | **25.32** |

*Taxon count includes all male and female fleas. **Count for flea species based on male fleas only. *^#^*Data based on 2019-2020 samples only. ^p^Predatory feeding.

**Supplementary Table 5.** Life stages and infestation parameters of epifaunistic species recorded on *Mastomys coucha* (n = 215) across a wildlife-human/domestic animal interface in Mpumalanga, South Africa, 2014-2020.

| Taxa | Total no. of individuals | Adults (%) | Larvae (%) | Nymphs (%) | Adult Male (%) | Adult Female (%) | Mean Abundance (±SE) | Prevalence (%) |
| --- | --- | --- | --- | --- | --- | --- | --- | --- |
| **Fleas*** | **222** | **100** | - | - | **49.1** | **50.9** | **1.03 (±0.14)** | **35.81** |
| *Echidnophaga gallinacea*** | 1 | 100 | - | - | - | - | - | 0.47 |
| *Listropsylla prominens*** | 8 | 100 | - | - | - | - | 0.04 (±0.01) | 3.26 |
| *Pariodontis* sp.** | 1 | 100 | - | - | - | - | - | 0.47 |
| *Xenopsylla bechuanae*** | 5 | 100 | - | - | - | - | 0.02 (±0.01) | 1.86 |
| *X. brasiliensis*** | 53 | 100 | - | - | - | - | 0.25 (±0.06) | 13.02 |
| *X. frayi*** | 41 | 100 | - | - | - | - | 0.19 (±0.04) | 13.49 |
| **Lice** | **2146** | **76.93** | - | **23.07** | **42.34** | **57.66** | **9.98 (±1.23)** | **54.42** |
| *Hoplopleura intermedia* | 2119 | 77.34 | - | 22.65 | 42.40 | 57.60 | 9.86 (±1.23) | 53.49 |
| *Polyplax biseriata* | 27 | 44.44 | - | 55.56 | 33.33 | 66.67 | 0.13 (±0.10) | 0.93 |
| **Ticks** | **144** | **-** | **61.81** | **38.19** | **-** | **-** | **0.67 (±0.18)** | **22.79** |
| *Dermacentor rhinocerinus* | 6 | - | 0 | 100 | - | - | 0.03 (±0.02) | 1.4 |
| *Haemaphysalis leachi/elliptica* group | 34 | - | 88.24 | 11.76 | - | - | 0.16 (±0.10) | 5.12 |
| *Hyalomma truncatum* | 9 | - | 88.89 | 11.11 | - | - | 0.04 (±0.02) | 3.26 |
| *Rhipicephalus follis/simus* group | 95 | - | 53.68 | 46.32 | - | - | 0.44 (±0.12) | 16.28 |
| **Mites** *^#^* | **2855** | **66.06** | **0.11** | **38.84** | **38.12** | **61.88** | **13.28 (±1.84)** | **92.5** |
| Ameroseiidae sp. | 1 | 100 | 0 | 0 | 0 | 100 | - | 0.83 |
| *Androlaelaps marshalli* | 11 | 81.82 | 9.09 | 9.09 | 0 | 100 | 0.05 (±0.03) | 5.83 |
| *A. oliffi* | 14 | 57.14 | 0 | 42.86 | 75 | 25 | 0.07 (±0.09) | 0.83 |
| *A. zuluensis* | 2 | 100 | 0 | 0 | 0 | 100 | 0.01 (±0.01) | 1.67 |
| *Austromyobia forcipifer* | 1 | 0 | 0 | 1 | 0 | 0 | 0 (±0.01) | 0.83 |
| *Cheyletus zumpti* ^p^ | 5 | 100 | 0 | 0 | 0 | 100 | 0.02 (±0.01) | 2.5 |
| *Laelaps giganteus* | 1 | 100 | 0 | 0 | 0 | 100 | - | 0.83 |
| *L. liberiensis* | 2761 | 65.23 | 0.07 | 34.70 | 39.42 | 60.58 | 12.84 (±1.82) | 89.17 |
| *L. muricola* | 47 | 100 | 0 | 0 | 4.26 | 95.74 | 0.22 (±0.08) | 20.83 |
| *Listriohoroides (A.) mastomys* | 8 | 100 | 0 | 0 | 12.5 | 87.5 | 0.04 (±0.04) | 1.67 |
| *Pachylaelaps* sp. ^p^ | 1 | 100 | 0 | 0 | 0 | 100 | 0 (±0.01) | 0.83 |
| Uropodoidae sp. ^p^ | 3 | 100 | 0 | 0 | 0 | 100 | 0.12 (±0.01) | 2.5 |
| **Chiggers** | - | - | - | - | - | - | - | **45** |

*Taxon count includes all male and female fleas. **Count for flea species based on male fleas only. *^#^*Data based on 2019-2020 samples only. ^p^Predatory feeding.

**Supplementary Table 6.** Species, prevalence and parasitope preference of chigger species recorded on *Mastomys natalensis* (n = 237) and *Mastomys coucha* (n = 120) across a wildlife-human/domestic animal interface in Mpumalanga, South Africa, 2019-2020.

|  | ***M. natalensis*** | | ***M. coucha*** | |
| --- | --- | --- | --- | --- |
| **Chigger species** | **Prevalence (%)** | **Parasitope** | **Prevalence (%)** | **Parasitope** |
| *Ascoschoengastia ueckermanni* | 0.84 | pinna | 1.67 | pinna |
| *Gahrliepia nana* | 0.42 | pinna body | 5.00 | pinna, tail base, hind leg |
| *Hypotrombidium clamatori* | 0.84 | pinna | 0.83 | pinna |
| *Microtrombicula mastomyia* | 22.36 | pinna | 36.67 | pinna, hind leg, scrotum |
| *Schoutedenichia dutoiti* | 4.22 | pinna | 0.83 | pinna |
| *S. lumsdeni* | 2.11 | pinna | 15.00 | pinna |
| *S. morosi* | 0.42 | pinna | - | - |
| *Susa hexasternalaea* | 0.42 | pinna | - | - |
